# Comparison of machine-learning algorithms to build a predictive model for detecting undiagnosed diabetes - ELSA-Brasil: accuracy study: Comparação de algoritmos de aprendizagem de máquina para construir um modelo preditivo para detecção de diabetes não diagnosticada - ELSA-Brasil: estudo de acurácia

**DOI:** 10.1590/1516-3180.2016.0309010217

**Published:** 2017-04-03

**Authors:** André Rodrigues Olivera, Valter Roesler, Cirano Iochpe, Maria Inês Schmidt, Álvaro Vigo, Sandhi Maria Barreto, Bruce Bartholow Duncan

**Affiliations:** I MSc. IT Analyst, Postgraduate Computing Program, Universidade Federal do Rio Grande do Sul (UFRGS), Porto Alegre (RS), Brazil.; II PhD. Professor, Postgraduate Computing Program, Universidade Federal do Rio Grande do Sul (UFRGS), Porto Alegre (RS), Brazil.; III PhD. Professor, Postgraduate Epidemiology Program and Hospital de Clínicas, Universidade Federal do Rio Grande do Sul (UFRGS), Porto Alegre (RS), Brazil.; IV PhD. Professor, Postgraduate Epidemiology Program, Universidade Federal do Rio Grande do Sul (UFRGS), Porto Alegre (RS), Brazil.; V PhD. Professor, Department of Social and Preventive Medicine & Postgraduate Program in Public Health, Universidade Federal de Minas Gerais (UFMG), Belo Horizonte (MG), Brazil.

**Keywords:** Supervised machine learning, Decision support techniques, Data mining, Models, statistical, Diabetes mellitus, type 2

## Abstract

**CONTEXT AND OBJECTIVE:**

Type 2 diabetes is a chronic disease associated with a wide range of serious health complications that have a major impact on overall health. The aims here were to develop and validate predictive models for detecting undiagnosed diabetes using data from the Longitudinal Study of Adult Health (ELSA-Brasil) and to compare the performance of different machine-learning algorithms in this task.

**DESIGN AND SETTING:**

Comparison of machine-learning algorithms to develop predictive models using data from ELSA-Brasil.

**METHODS:**

After selecting a subset of 27 candidate variables from the literature, models were built and validated in four sequential steps: (i) parameter tuning with tenfold cross-validation, repeated three times; (ii) automatic variable selection using forward selection, a wrapper strategy with four different machine-learning algorithms and tenfold cross-validation (repeated three times), to evaluate each subset of variables; (iii) error estimation of model parameters with tenfold cross-validation, repeated ten times; and (iv) generalization testing on an independent dataset. The models were created with the following machine-learning algorithms: logistic regression, artificial neural network, naïve Bayes, K-nearest neighbor and random forest.

**RESULTS:**

The best models were created using artificial neural networks and logistic regression. ­These achieved mean areas under the curve of, respectively, 75.24% and 74.98% in the error estimation step and 74.17% and 74.41% in the generalization testing step.

**CONCLUSION:**

Most of the predictive models produced similar results, and demonstrated the feasibility of identifying individuals with highest probability of having undiagnosed diabetes, through easily-obtained clinical data.

## RESUMO

**CONTEXTO E OBJETIVO:**

Diabetes tipo 2 é uma doença crônica associada a graves complicações de saúde, causando grande impacto na saúde global. O objetivo foi desenvolver e validar modelos preditivos para detectar diabetes não diagnosticada utilizando dados do Estudo Longitudinal de Saúde do Adulto (ELSA-Brasil) e comparar o desempenho de diferentes algoritmos de aprendizagem de máquina.

**TIPO DE ESTUDO E LOCAL:**

Comparação de algoritmos de aprendizagem de máquina para o desenvolvimento de modelos preditivos utilizando dados do ELSA-Brasil.

**MÉTODOS:**

Após selecionar 27 variáveis candidatas a partir da literatura, modelos foram construídos e validados em 4 etapas sequenciais: (i) afinação de parâmetros com validação cruzada *(10-fold cross-validation)*; (ii) seleção automática de variáveis utilizando seleção progressiva, estratégia *“wrapper”* com quatro algoritmos de aprendizagem de máquina distintos e validação cruzada para avaliar cada subconjunto de variáveis; (iii) estimação de erros dos parâmetros dos modelos com validação cruzada; e (iv) teste de generalização em um conjunto de dados independente. Os modelos foram criados com os seguintes algoritmos de aprendizagem de máquina: regressão logística, redes neurais artificiais, *naïve* Bayes, K vizinhos mais próximos e floresta aleatória.

**RESULTADOS:**

Os melhores modelos foram criados utilizando redes neurais artificiais e regressão logística alcançando, respectivamente, 75,24% e 74,98% de média de área sob a curva na etapa de estimação de erros e 74,17% e 74,41% na etapa de teste de generalização.

**CONCLUSÃO:**

A maioria dos modelos preditivos produziu resultados semelhantes e demonstrou a viabilidade de identificar aqueles com maior probabilidade de ter diabetes não diagnosticada com dados clínicos facilmente obtidos.

## INTRODUCTION

Type 2 diabetes is a chronic disease characterized by the body’s inability to efficiently metabolize glucose, which increases blood glucose levels and leads to hyperglycemia. This condition is associated with a wide range of serious health complications affecting the renal, neurological, cardiac and vascular systems, and it has a major impact on overall health and healthcare costs.^1^

Recent studies have estimated that around 415 million people have diabetes, and that the number of cases may increase to 642 million by 2040. In addition, approximately half of these individuals are not aware of their condition, which may further intensify the negative consequences of the disease. Diabetes was the main cause of death of nearly five million people in 2015, and it has been estimated that by 2030 it will become the seventh largest cause of death worldwide.^2^^,^^3^^,^^4^

It is believed that diabetes, like other noncommunicable chronic diseases, is mainly caused by behavioral factors such as poor diet and physical inactivity. Early interventions aimed towards creating lifestyle changes, with or without associated pharmacological therapies, have been proven effective in delaying or preventing type 2 diabetes and its complications. This has led many countries to invest in national programs to prevent this disease. To reduce costs and amplify the results, population-level interventions need to be combined with interventions that are directed towards individuals who are at high risk of developing or already having diabetes,^5^ so as to focus interventions, at the individual patient level, on those for whom they are most appropriate.

To this end, over recent years, a series of clinical prediction rules have been developed to identify individuals with unknown diabetes or those at high risk of developing diabetes.^5^^,^^6^^,^^7^^,^^8^^,^^9^ However, few of these rules have been drawn up using the most recently developed machine-learning techniques, which potentially have the ability to produce algorithms of greater predictive ability than those developed through the technique most commonly used to date, i.e. multiple logistic regression.

## OBJECTIVE

This paper presents the development and comparison of predictive models created from different machine-learning techniques with the aim of detecting undiagnosed type 2 diabetes, using baseline data from the Longitudinal Study of Adult Health (ELSA-Brasil).

## METHODS

These analyses were performed on data from the baseline survey (2008-2010) of ELSA-Brasil, a multicenter cohort study that had the main aim of investigating multiple factors relating to adult health conditions, including diabetes and cardiovascular diseases. The study enrolled 15,105 public servants aged between 35 and 74, at six public higher-education and research institutions in different regions of Brazil between 2008 and 2010, as has been previously reported in greater detail.^10^^,^^11^ The institutional review boards of the six institutions at which the study was conducted gave their approval, and written informed consent was obtained from all participants.

All analyses were performed using R version 3.2.3. The source codes used in the analysis are freely available.

### Dataset and preliminary variable selection

Data from the ELSA study baseline were used to create the predictive models. At this baseline, the 15,105 participants were evaluated through interviews, clinical examinations and laboratory tests. The interviews addressed educational achievement; characteristics and composition of home and family; dietary habits; alcohol drinking habits; smoking habits; presence of dyslipidemia or hypertension; physical activity at leisure; sleep quality; medical history; and medication use, among other topics. The examinations involved anthropometric measurements and blood and urine tests, among others. The study generated more than 1500 variables for each participant at baseline, as described previously.^10^^,^^11^

A total of 1,473 participants were excluded from the present analyses because they had self-reported diabetes. Another three participants were excluded because some information required for characterizing undiagnosed diabetes was missing. An additional 1,182 participants (8.7%) were excluded from the analyses because data relating to other variables were missing. Among the remaining 12,447 participants, 1,359 (11.0%) had undiagnosed diabetes.

Undiagnosed diabetes was considered present when, in the absence of a self-report of diabetes or use of anti-diabetic medication, participants had fasting glucose levels ≥ 126 mg/dl, glucose levels ≥ 200 mg/dl two hours after a standard 75 g glucose load or had glycated hemoglobin (HbA1c) ≥ 6.5%.

Through procuring variables in the ELSA dataset that were similar to those investigated in previously published predictive models for detecting diabetes or in situations of high risk of developing diabetes, we selected 27 diabetes risk factors for analysis. Any variables that implied additional costs beyond those of filling out a questionnaire and performing basic anthropometry, such as clinical or laboratory tests, were excluded so that the model obtained could be applied using a straightforward survey and simple anthropometric measurements. The final variable subset was validated by experts, and this resulted in the subset of 27 candidate variables described in Table 1 and Table 2. Table 2 also presents the analysis target variable of prevalent diabetes “a_dm”.

**Table 1:**
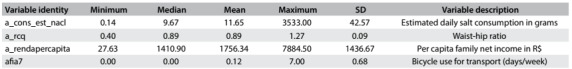
Numerical variables

SD = standard deviation.

**Table 2:**
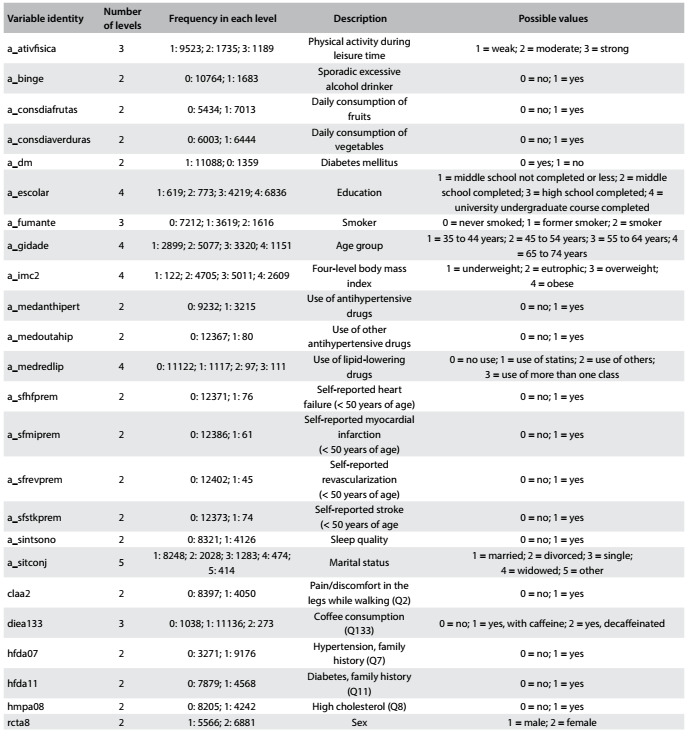
Categorical variables, including the target variable “a_dm”

The original dataset was randomly divided into two parts in the ratio of 70:30. The first part (training/validation dataset) was used for parameter and cutoff tuning, automatic variable selection and error estimation using cross-validation; the second part (test dataset) was used for generalization tests. The models created were evaluated in terms of area under the receiver operating characteristic curve (AUC), sensitivity, specificity and balanced accuracy (arithmetic mean of sensitivity and specificity). The machine-learning algorithm families of logistic regression, artificial neural network (multilayer perceptron/backpropagation), Bayesian network (naïve Bayes classifier), instance-based learning (K-nearest neighbor) and ensemble (random forest) were used.

### Machine-learning algorithms

The machine-learning algorithms are briefly described below:

*Logistic regression*^12^ is a well-established classification technique that is widely used in epidemiological studies. It is generally used as a reference, in comparison with other techniques for analyzing medical data.

*Multilayer perceptron/backpropagation*^13^ is the principal artificial neural network algorithm. When there is no hidden layer on the network, this algorithm is equivalent to logistic regression, but it can solve more difficult problems with more complex network architectures. The price of using complex architectures is that it produces models that are more difficult to interpret. Additionally, it can be computationally expensive.

*Naïve Bayes classifier*^14^ is a type of Bayesian network that, despite enormous simplicity, is able to create models with high predictive power. The algorithm works well with heterogeneous data types and also with missing values, because of the independent treatment of each predictor variable for model construction.

*K-nearest neighbor* (*instance-based learning*)^15^ is a classical instance-based learning algorithm in which a new case is classified based on the known class of the nearest neighbor, by means of a majority vote. This type of algorithm is also called lazy learning because there is no model building step and the entire computing procedure (i.e. the search for the nearest neighbor) is performed directly during the prediction. All the cases (training/validation dataset) need to be available during the prediction.

*Random forest*^16^ is a machine-learning algorithm from the “ensemble” family of algorithms,^17^ which creates multiple models (called weak learners) and combines them to make a decision, in order to increase the prediction accuracy. The main idea of this technique is to build a “forest” of random decision “trees” and use them to classify a new case. Each tree is generated using a random variable subset from the candidate’s predictor variables and a random subset of data, generated by means of bootstrap. This algorithm also can be used to estimate variable relevance.

### Data preparation

#### Standardization of numerical variables

Transformation between different data types (categorical or numerical) was performed by means of binarization or discretization, when necessary. In binarization, a categorical variable with *n* levels is transformed into *n* - 1 dummy variables that have values equal to “1” when the case belongs to the level represented by the dummy variable or “0” otherwise.

In discretization, a numerical variable is transformed into a categorical variable by defining a set of cutoff points for that variable, such that the ranges of values between the cutoff points correspond to the levels of the categorical variable. The Ameva algorithm^18^ was used to find the best cutoff points for each numerical variable.

#### General process of model construction and evaluation

The models were built, evaluated and compared using four sequential steps:

1. Parameter tuning;
2. Automatic variable selection;
3. Error estimation; and
4. Generalization testing in an independent dataset.

**Figure 1:**
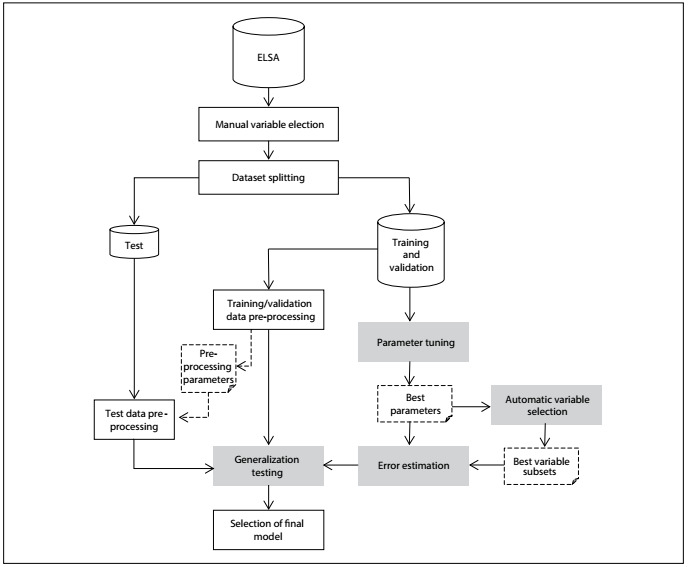
General process of model construction and evaluation.

The complete process is depicted in Figure 1. First, manual variable preselection was performed as described above (“Manual Variable Selection” box in the Figure). After that, 30% of the dataset (“Test” dataset in the Figure), containing 3,709 complete cases, was separated for generalization testing, while the other part (“Training/Validation” dataset in the Figure), containing 8,738 complete cases, was used as the dataset for the first three steps of the process.

The first step in model building (“Parameter Tuning” box in Figure 1) evaluated each machine-learning algorithm with different sets of configurable parameters of the algorithm by means of tenfold cross-validation, repeated three times. In tenfold cross-validation, the dataset (training/validation) is divided into ten parts, of which nine are used for training (selecting) a model and the tenth for validation of this model. This process is repeated to calculate the validation measurements, such as AUC, while varying the part of the dataset used for validation each time. Finally, the mean of the validation measurements across repeats is calculated. The results from this step (“Best Parameters” item in the Figure), containing the best parameters and cutoffs for classification for each algorithm, were used in the next steps.

The second step (“Automatic Variable Selection” box in Figure 1) generated four different variable subsets using different algorithms and cross-validation (using only the best settings found in the preceding step), with the wrapper strategy and a forward selection search for automatic variable selection. The best variable subsets found in this step (“Best Variable Subsets” item in Figure 1) were used in the next steps.

The third step (“Error Estimation” box in Figure 1) used cross-validation to obtain more reliable estimates of the performance of different learning schemes, using the best settings and subsets obtained in the preceding steps.

Finally, the last step (“Generalization Testing” box in Figure 1) evaluated models using only the learning scheme that obtained the best performance for each algorithm in the test dataset that had not previously been used.

The following sections describe each step in more details.

### Parameter tuning

This first step in model building evaluated each algorithm with different parameter configurations to find out which parameter configuration produced the best results for each algorithm and data type conversion used. The parameters tested for each algorithm are listed in Table 3.

**Table 3:**
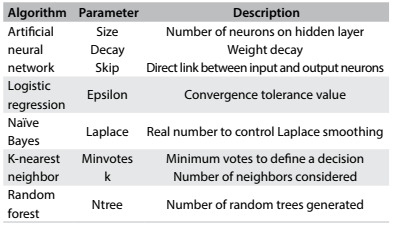
Parameters analyzed in parameter tuning

Because of the wide range of possible values for the parameters, a search strategy was adopted. At first, a limited set of values for each parameter was selected, and each combination of parameters was evaluated by means of tenfold cross-validation, repeated three times, thus generating 30 models. Each instance of machine learning was tested with and without data discretization. The results from the 30 models generated in each test were averaged. The parameter configuration that produced the best mean AUC was chosen. Moreover, a set of different cutoffs (predefined by the analyst) to generate the classification was evaluated to find out which produced the best classification on average between the 30 models in terms of balanced accuracy.

After that, the results were analyzed and, when necessary, new parameter values and/or cutoff points were selected for new tests. In this case, the new values were selected around the values from which the best results had been obtained up to that moment. The idea was to start testing a sparse range of values and then decrease the granularity of the values in order to avoid trying values that were very likely to produce poor results. This search stopped when there was no increase in the predictive power of the models that had been created using the specific machine-learning algorithm and data type conversion evaluated.

### Automatic variable selection

The automatic variable selection step had the aim of finding subsets from the 27 candidate variables that could increase the performance of the predictive models, compared with other models created using different sets of candidate variables.

These subsets of variables were generated using the wrapper strategy.^19^ In this strategy, models are created and evaluated by means of a machine-learning algorithm and a validation method, such as cross-validation, using different subsets of variables. The subset from which the best performance is achieved, in terms of a criterion such as AUC, is chosen as the best subset. Because of the large number of possible subsets, a heuristic search was used to generate the variable subset candidates that were more likely to create better models, thereby optimizing the process. The main advantage of this method compared with other strategies is that it evaluates multiple variables together. The drawback is that, because it depends on a machine-learning algorithm to create/evaluate models, it is possible that the subset of variables that produces the best results using one algorithm can produce bad results when using another algorithm or another parameter setting for the same algorithm.

Four machine-learning algorithms were used: logistic regression, artificial neural network, K-nearest neighbor and naïve Bayes classifier. The random forest algorithm was not included because it already performs an embedded variable selection. The forward selection search strategy was used in modeling because it tends to generate smaller subsets. The same validation technique (tenfold cross-validation repeated three times), decision criterion (mean AUC) and dataset partition that had been used in the parameter tuning step were used again in this step. The best parameter settings obtained in the parameter tuning step were used to configure the parameters of the algorithms for this step. Each machine-learning technique generated a distinct subset of variables. The subsets thus generated were used in the next step.

### Error estimation

The error estimation step evaluated each machine-learning algorithm using the parameters obtained in the first step and the subsets generated in the second step, in addition to the original variable subset containing all the candidate variables. This step also served to evaluate the use of discretization. The evaluation was done through tenfold cross-validation, which was repeated ten times to get more reliable prediction performance estimates.

### Generalization testing

Finally, one model was generated from the training/validation dataset for each algorithm, using the best results from the preceding step. These best models were then evaluated (hold-out evaluation) in the test set, since this generalization testing has the aim of evaluating model behavior when faced with data that was not used in its creation. The results from this evaluation serve as a quality measurement for these models.

### Development of an equation for application of the results

The model with best results from generalization testing was used to create a web tool to apply the questionnaire in practice. The prediction from the logistic regression model for any given individual is calculated by multiplying that individual’s value for each variable in the model by the coefficient derived from the model for that variable, and then summing the results and transforming this sum into a probability of undiagnosed diabetes using the logistic function. If this probability is above the predetermined cutoff (here, 11%), the individual is classified as positive (at high risk of undiagnosed diabetes); or otherwise, as negative.

## RESULTS

### Study sample

Among the 12,447 ELSA participants included in this study, 5,566 (44.67%) were men. The participants were between 35 and 74 years old; the largest proportion (5,077) was in the group between 45 and 54 years old; 6,836 (54.92%) had a complete university education or more; 5,011 (40.26%) were overweight and 2,609 (20.96%) were obese. Using the World Health Organization definition (fasting glucose ≥ 110 mg/dl and/or 2 hour postload glucose ≥ 140 mg/dl), 5,539 (44.5%) presented intermediate hyperglycemia. Other details about the study sample can be found in Table 1 and Table 2.

### Parameter tuning

The best parameter configuration for each data type conversion of each algorithm is depicted in Table 4.

**Table 4:**
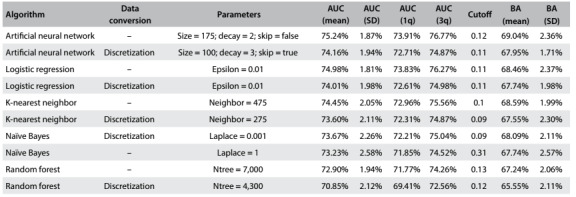
Results from parameter tuning

AUC = area under the ROC curve; SD = standard deviation; 1q/3q = first and third quartiles; BA = balanced accuracy.

The first and second columns of Table 4 present the name of the algorithm and whether discretization was used, respectively. The third column shows the values of the parameter configuration that provided the best result for the machine-learning algorithm and data type conversion of each row. The next four columns present basic statistics (mean, standard deviation, first and third quartiles and cutoff points, respectively) of the AUC obtained in the cross-validation. The eighth column shows the cutoff that provided the mean best balanced accuracy (BA) and the last two columns shows the mean balanced accuracy and its standard deviation.

Table 4 shows each machine-learning algorithm with its different data type conversions, sorted in descending order in terms of AUC and balanced accuracy for each algorithm and data type conversion.

Although defining which algorithms produce better results was not the objective of this step, it was possible to gain an initial insight into their predictive powers. In this regard, the best results were produced by artificial neural networks and logistic regression with mean AUC of 75.24% (row 1) and 74.98% (row 3), respectively.

Table 4 also shows the impact in terms of performance, when discretization was used in each machine-learning algorithm. For example, performance decreased (around 1% overall and almost 3% in the case of random forest) when the data were discretized in the models generated by all the algorithms except naïve Bayes. In general, the performance behavior of the machine-learning algorithms and conversion remained similar for the next steps.

Another result that can be seen in most cases is the impact on the choice of the parameter settings, caused by the conversion used. For example, the best performance of the artificial neural network algorithm was achieved without data conversion and with size = 175 (i.e. 175 neurons in the hidden layer). However, when discretization was used, the best parameter setting changed to size = 100.

The best parameter setting achieved was used to configure the five algorithms used for the automatic variable selection step, as well as in further steps.

### Results from automatic variable selection

The automatic variable selection step generated four distinct subsets of variables as shown in Table 5 (rows 1 to 4): *lr-fs,* created with logistic regression (*fs* in the name stands for “forward selection”); *ann-fs,* created with an artificial neural network; *knn-fs,* created with K-nearest neighbor; and *nb-fs,* created with a naïve Bayes algorithm.

**Table 5:**
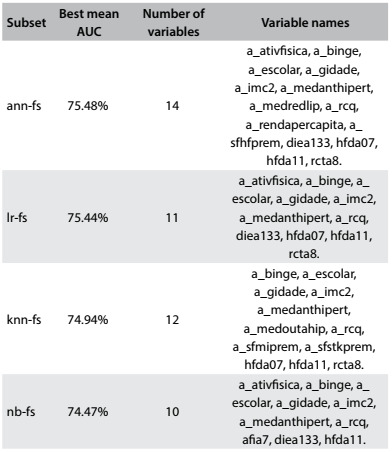
Variable subsets generated in automatic variable selection

The first column of Table 5 shows the identifier name of the subset, the second column presents the AUC achieved by the variable subset that was chosen for each algorithm, the third shows the number of variables of each subset and the fourth presents these variable names.

The dataset partitions used for this step were the same as used in the parameter tuning step. Thus, it is possible to gain an insight into the performance improvement in terms of AUC when using a variable subset instead of using all the variables from the original dataset. Furthermore, merely the fact that a smaller subset was used to create the models is already an advantage because this makes the model and its application much simpler.

Because of the nature of the wrapper strategy, it can be expected that each machine-learning algorithm will present better results when using the variable subset created by the algorithm itself. However, in the next step all the subsets were tested with all the algorithms.

### Results from error estimation

The aim of this step was to obtain more reliable error estimates regarding algorithm performance. For this reason, 10 repetitions were used instead of 3, for the repeated tenfold cross-validation, thus generating 100 models instead of 30 for each test.

The machine-learning algorithms were tested using the best parameters found in the first step (depicted in Table 4), with the variable subsets generated in the second step (described in Table 5), as well as with the original set of variables. Performance was tested with and without discretization.

Table 6 describes the best results obtained for each machine-learning algorithm, variable subset and data conversion used. Respectively, the columns represent the name of machine-learning algorithm used; data type conversion; variable subset; AUC mean, standard deviation (SD) and first and third quartiles achieved in cross-validation; and mean and standard deviation of the balanced accuracy (BA).

**Table 6:**
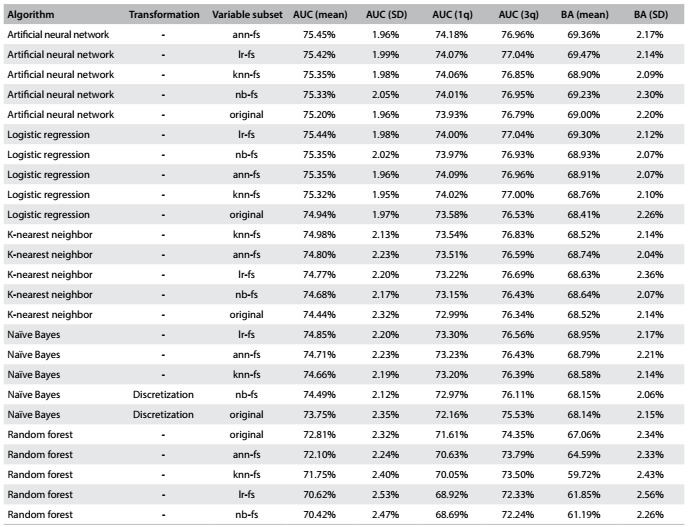
Error estimation results

AUC = area under the ROC curve; SD = standard deviation; 1q/3q = first and third quartiles; BA = balanced accuracy.

Like in the results from the parameter tuning step, the artificial neural network algorithm and logistic regression achieved the best results. Without data conversion, these algorithms produced similar models, with AUC of 75.45% (row 1) and 75.44% (row 4), respectively, each using the variable subset generated with its own algorithm, as expected. K-nearest neighbor and naïve Bayes also reached good results, with AUC of close to 75%. The best results with the naïve Bayes classifier were obtained using a subset of variables other than *nb-fs.* This was possible because the variable subset search with this algorithm used discretized data following the best results from parameter tuning, while the best result in the current phase was without variable transformation.

Finally, as in the parameter tuning step, random forest produced the worst results. Independent of the subset of variables, this algorithm showed a worse yield in terms of mean AUC.

Table 6 also shows the impact of using a specific variable subset, compared with the best results obtained from the models generated using the original variable set. This difference is very small: around 0.25% better using the variable subset instead of all the original variables for the artificial neural network models. The results obtained with a subset of variables were slightly better (around 0.5%) than the original with logistic regression and K-nearest neighbor models. The best naïve Bayes classifier model result from using a variable subset was more than 1% better than the best result from using all the variables. Finally, random forest models produced the best results using all of the available variables.

### Results from generalization testing

In generalization testing, the best learning scheme (which includes data type conversion used, parameter setting, classification cutoff and variable subset) found for each algorithm in the preceding step was evaluated in the test dataset, which had been separated at the beginning of the process and had not been used until this step.

Table 7 shows the best results obtained in the error estimation phase together with the results obtained in generalization testing.

**Table 7:**
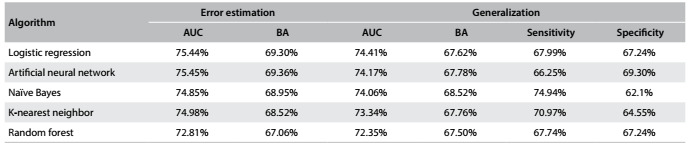
Generalization testing results compared with those of the error estimation step

AUC = area under the ROC curve; BA = balanced accuracy.

All the algorithms maintained good performance in generalization testing. The biggest loss of performance in relation to the error estimate step, as assessed from changes in the AUC, was 1.64% for the K-nearest neighbor algorithm. The artificial neural network, logistic regression and naïve Bayes had performance losses of 1.30%, 1.03% and 0.80%, respectively. The least loss in generalization testing was 0.458%, achieved by the random forest algorithm, which produced the worst performance in terms of AUC of all the algorithms. Nevertheless, the worst result was an AUC of 72.35%.

Since the best result from this step in terms of AUC (74.41%) was obtained using logistic regression, and given the easy interpretation and applicability of this model, logistic regression was chosen to be used to create the diabetes risk assessment tool.

### Web tool proposed for detecting undiagnosed diabetes

**Figure 2:**
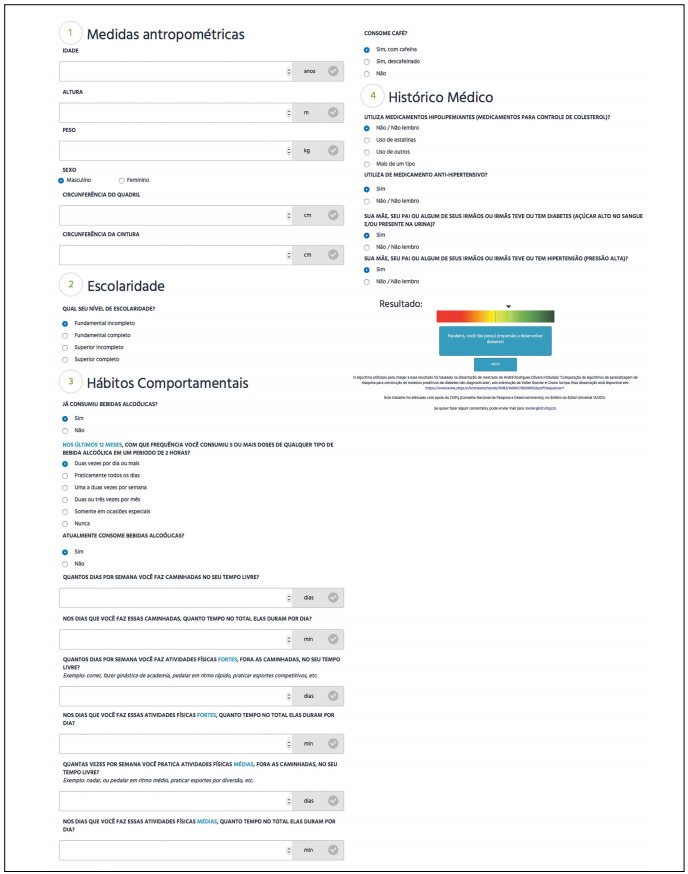
Prototype for a web interface for the risk equation.

Finally, the model generated using the logistic regression algorithm in the generalization test was selected to build a web tool for detecting undiagnosed diabetes. This model produced sensitivity of 68% and specificity of 67.2%. The prototype interface of the tool is shown in Figure 2. Since the model was constructed and probably would be used in Brazil, the tool was created in Portuguese.

The final coefficients of the equation generated are described in Table 8.

**Table 8:**
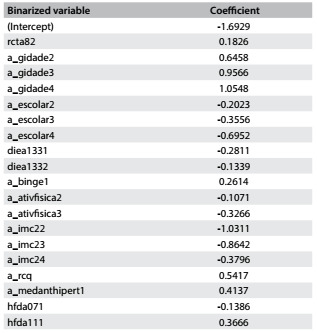
Coefficients from logistic regression model

New cases can be classified using this model, as follows:

1. Standardize the value of the only numerical variable (a_rcq) by subtracting the training mean (0.8889311) from the value and dividing the result by the training standard deviation (0.08615528).
2. Binarize the categorical variables;
3. Calculate the sum of the variables created in the preceding steps using the coefficients from Table 8;
4. Add to this sum the value of the intercept term, described in the first row of Table 8;
5. Calculate the probability of undiagnosed diabetes for a given individual = 1/(1+e^-x^), where x equals the sum resulting from the preceding steps.

If the probability is greater than 0.11, then classify the individual as presenting high risk of having undiagnosed diabetes; otherwise, classify the individual as presenting low risk.

## DISCUSSION

We created predictive models for detecting undiagnosed diabetes using data from the ELSA study with different machine-learning algorithms. The best results were achieved through both an artificial neural network and logistic regression, with no relevant difference between them.

Generally, most of the algorithms used achieved mean AUCs greater than 70%. The best algorithm (logistic regression) produced an AUC of 74.4%. Since these test dataset values are superior to the AUCs of several other scores that were previously validated in other populations,^20^ this score shows potential for use in practice.

The generalization testing showed the results from asking a population similar to that of ELSA some simple questions. Out of 403 individuals from the testing dataset who had diabetes and did not know about their condition, 274 were identified as positive cases (68.0% sensitivity) using the model generated through the logistic regression algorithm. The web tool prototype for detecting undiagnosed diabetes could be refined for use in Brazil.

The methods and concepts for building predictive models for use in healthcare, as well as the challenges and difficulties faced when analyzing healthcare data, have been well described.^17^^,^^18^^,^^19^^,^^20^^,^^21^^,^^22^^,^^23^ Many groups have published predictive models for detecting undiagnosed diabetes. Although several groups have reported AUCs above 0.80, these values generally reduce to < 0.70 when tested on independent samples.^20^ Differences in predictive power across studies can be ascribed to different characteristics relating to the different datasets, and to different techniques and methods for building and evaluating the models. The characteristics that may vary across studies include the definition of the target variable, model objectives and candidate variables, among others. These models are generally constructed using conventional statistical techniques such as logistic regression and Cox regression. Systematic reviews^5^^,^^16^^,^^24^^,^^25^^,^^26^ present several such studies: some, like ours, have focused on predicting undiagnosed diabetes; while others have focused on individuals at high risk of developing incident diabetes.

Use of machine-learning techniques is still new in this field.^27^^,^^28^^,^^29^ The main studies have compared the results obtained through using a specific technique with the results obtained through logistic regression. One report^30^ described creation of pre-diabetes risk models using an artificial neural network and support-vector machines that were applied to data from 4,685 participants in the Korean National Health and Nutrition Examination Survey (KNHANES), collected between 2010 and 2011. In comparison with results^31^ from logistic regression on the same dataset, the models created using support-vector machines and an artificial neural network produced slightly better results.

Two other reports^32^^,^^33^ also compared artificial neural networks with logistic regression for creating predictive diabetes models. In the first, models created using artificial neural networks on data from 8,640 rural Chinese adults (760 of them with diabetes) produced better results (AUC = 89.1% ± 1.5%) than models created using logistic regression (AUC = 74.4% ± 2.1%). In the second, a radial basis function artificial neural network that was applied to data from 200 people (100 cases with diabetes and 100 with pre-diabetes) at 17 rural healthcare centers in the municipality of Kermanshah, Iran, showed better results than logistic regression and discriminant analysis, for identifying those with diabetes. Another study^34^ comparing diabetes models created using data from 2,955 women and 2,915 men in the Korean Health and Genome Epidemiology Study (KHGES) showed similar results from logistic regression and naïve Bayes, although naïve Bayes showed better results with unbalanced datasets. Finally, another study^35^ used data from 6,647 participants (with 729 positive cases) in the Tehran Lipid and Glucose Study (TLGS) and created models with decision trees reaching 31.1% sensitivity and 97.9% specificity (balanced accuracy was around 64.5%),^36^ for detecting increased blood glucose levels.

In summary, use of machine-learning techniques may prove to be a viable alternative for building predictive diabetes models, often with good results, but rarely with notably superior results, compared with the conventional statistical technique of logistic regression.

## CONCLUSION

Comparison between different techniques showed that all of them produced quite similar results from the same dataset used, thus demonstrating the feasibility of detecting undiagnosed diabetes through easily-obtained clinical data. The predictive algorithm for identifying individuals at high risk of having undiagnosed diabetes - based only on self-reported information from participants in ELSA-Brasil, from which the highest AUC (0.74) was obtained when tested on a part of the sample that had not been used for its derivation - was a logistic regression equation. However, the machine-learning techniques of artificial neural network, naïve Bayes, k-nearest neighbor and random forest all produced AUCs that were similar or slightly smaller.
